# Larvicidal activity of *Stemona collinsiae* root extract against *Musca domestica* and *Chrysomya megacephala*

**DOI:** 10.1038/s41598-023-42500-8

**Published:** 2023-09-21

**Authors:** Aurapa Sakulpanich, Siriluck Attrapadung, Wandee Gritsanapan

**Affiliations:** 1 Division of Pharmaceutical Sciences, Faculty of Pharmacy, Thammasat University, Pathum Thani, Thailand; 2 Department of Medical Entomology, Faculty of Tropical Medicine, Mahidol University, Bangkok, Thailand; 3 Phyto Product Research, 165 Soi Suwandee 3, Rimklongprapa Road, Bangsue, 10800 Bangkok Thailand

**Keywords:** Natural products, Entomology

## Abstract

*Musca domestica* and *Chrysomya megacephala*, considered synanthropic insects, are medically important flies, as they transmit vector-borne diseases to humans and animals. In Thailand, *Stemona* (Stemonaceae) plants have been traditionally used as insecticides. This study was designed to determine the larvicidal activity of *S. collinsiae* root extract against *M. domestica* and *C. megacephala* larvae. A 70% ethanol crude extract from *S. collinsiae* roots was tested against the third-instar larvae of both species using direct and indirect contact methods. The development and mortality rates of the insects were observed, and the LC values were calculated. The extract caused irregular development in both species, shown as segmental puparia that could not emerge as adult flies. The LC_50_ values of the extract against *M. domestica* tested by direct and indirect contact methods were 0.0064 ± 0.0005 mg/larva and 0.0165 ± 0.0002 mg/cm^2^/larva, respectively. In the case of *C. megacephala*, the LC_50_ value determined by the indirect contact method was 1.0500 ± 0.0001 mg/cm^2^/larva. The ethanolic root extract of *S. collinsiae* was able to kill the larvae of both species after dermal administration. It is of interest to develop *S. collinsiae* root extract as a natural fly control biopesticide.

## Introduction

According to indigenous Thai knowledge, *Stemona* plants have been used as pesticides and fly repellents. *Stemona* plants have been reported to have antitussive^1^, antifungal^2^, and insecticidal activities^3–5^. *Stemona* root extracts can kill neonate larvae and fifth-instar larvae of *Spodoptera littoralis* Boisduval. The root extract of *S. collinsiae* displays higher activity than the leaf extract and has the highest anti-feedant and growth inhibitory activities among *S. tuberosa*, *Aglaia edulis*, *A. odorata*, and pyrethrum extracts^6^. Furthermore, *S. collinsiae* root extract has shown insecticidal activity against *Plutella xylostella*^7^ and *Parasarcophaga ruficornis*^8^.

*M. domestica* and *C. megacephala* are considered to be medical flies because they are vectors of several diseases, such as cholera, dysentery, and food poisoning. Pathogenic parasites, bacteria, or viruses can be transmitted from flies to humans and animals by mechanical transmission and biological transmission^9–13^. Furthermore, fly larvae have been found to be a cause of myiasis in cattle^14^ and humans^15^. To protect humans and animals from disease, good hygiene and sanitation practices should be performed, and chemical insecticides should be used to eliminate flies. Chemical insecticides are widely used to control flies and can kill them in large numbers. Chemical insecticides are categorized as synthetic-chemical or natural insecticides. Naturally occurring insecticides such as nicotine and pyrethrin are sources of chemical insecticides. Some synthetic chemical insecticides are produced by mimicking natural insecticides. Imidacloprid mimics nicotine^16^, while pyrethroid pesticides are modified from the natural insecticide pyrethrin, which is found in chrysanthemum^17,18^. Insecticidal plants are recognized as biopesticides that may be produced from plants, microbes, or living microorganisms and are considered to be environmentally friendly with a short degradation time, low level of toxicity, and low impact on biodiversity^19,20^. *S. collinsiae* is an interesting plant and has been used as an insecticide for a long time. It presents good insecticidal activity and contains a high content of the insecticidal alkaloid didehydrostemofoline. The aim of this research is to detect the larvicidal activity of *S. collinsiae* root extract in third-instar larvae of *M. domestica* and *C. megacephala* using contact administration. The lethal concentrations (LC_50_ and LC_90_ values) of the extracts and the percent mortality were calculated. Signs of toxicity were observed, recorded and compared with the negative and positive control groups.

## Materials and methods

### Plant materials

*S. collinsiae* roots were obtained from an agriculturist who planted *S. collinsiae* for personal plantation use in Ubon Ratchathani, Thailand. The obtained *S. collinsiae* was used for research without commercial expectation. It was harvested between December 2014 and March 2015. *S. collinsiae* (root and aerial parts) was identified by a botanist and deposited at the Forest Herbarium, Department of National Park, Wildlife and Plant Conservation, Ministry of Natural Resources and Environments, Bangkok, with voucher specimens BKF No. 196976. Other voucher specimens (SC001WD) were deposited at Department of Pharmacognosy, Faculty of Pharmacy, Mahidol University, Thailand.

The fresh roots were cleaned with tap water, cut into small pieces, and dried in a hot air oven at 50 °C for 72 h. The dried roots were ground into powder and passed through a sieve (60 mesh). The powder was stored in an airtight amber container at 25 °C until use.

### Chemicals and reagents

Ethanol (95%) for extraction was purchased from Liquor Distillery Organization, Excise Department, Ministry of Finance, Thailand. The imidacloprid Pestanol® analytical standard was purchased from Sigma‒Aldrich® (Singapore). Deionized water was purified by Water Pro PS (Labconco, Missouri, USA). All reagents were of analytical grade.

### Preparation of the *S. collinsiae* root extract

The powdered roots of *S. collinsiae* were completely extracted using reflux extraction with 70% ethanol as the extractant. First, 70% ethanol was added to the flask containing the powdered roots. The flask was soaked in warm water (controlled at 60–70 °C) for 1 h. After 1 h, the liquid extract was filtered through Whatman^TM^ filter paper (GE healthcare UK Limited, China). The filtrate was kept in a glass bottle. Then, 70% ethanol was added to the residue of *S. collinsiae* again. Extraction was performed repeatedly until the alkaloids in the powdered roots of *S. collinsiae* were exhaustively extracted. Complete extraction was monitored by TLC and Dragendorff’s spray reagent. The occurrence of orange bands indicated the presence of alkaloids. The mobile phase was a mixture of dichloromethane, ethyl acetate, methanol, and 10% NH_4_OH at a ratio of 70:25:5:1, and the stationary phase was a silica gel GF_254_ TLC plate (Merck, Germany). Finally, the combined filtrate was concentrated using a rotary evaporator under reduced pressure and at 40 °C. The concentrated extract was evaporated to dryness in a hot water bath to yield a semisolid crude extract. The crude ethanol extract was kept in a glass bottle protected from light in a refrigerator at -20 °C for further experiments. The phytochemicals in the semisolid crude extract were repeatedly checked by TLC.

### Fly rearing (modified from^21^)

Adult-stage *M. domestica* were collected from a poultry farm in Chachoengsao Province, Thailand, while *C. megacephala* were collected from Wat Som Kliang in Nonthaburi Province, Thailand, by a sweeping net method. The species of flies were identified by an entomologist. Each species of adult flies was reared in entomology cages (30 × 30 × 30 cm) at the Faculty of Tropical Medicine, Mahidol University under the following conditions: 28–30 °C (65–70% humidity) and a 12:12 light/dark cycle. Water, milk powder, and granulated sugar were provided in the cage as the adult diet.

A fresh pig lung was purchased from a food market in Phran Nok Market in Bangkok, Thailand. Pieces of the fresh pig lung (250 g) were prepared in a plastic cup as the oviposition site and for the larvae diet. The oviposition site was checked daily. When groups of fly eggs were found, the plastic cup was separated from the entomology cage and placed into a plastic tank that had a dry area for third-instar larvae migration. The plastic cup and plastic tank used were designed according to the behavior of third-instar larvae that migrate from high humidity areas to dry areas; the third-instar larvae do not need food. The plastic tank was covered with a muslin cloth and then tied with a rubber band for protection from other insects. The diet of the larvae was pig lung, which was checked daily and added in sufficient amounts for the first- and second-instar larvae. The third-instar larvae that moved to the dry area were separated for further experiments.

The size, stage and morphological characteristics of the third-instar larvae (20) were detected under an SNZ745T stereomicroscope 10 × (Nikon, China) and Eclipse E200-LED microscope 4 × to 40 × (Nikon, China) with a microscope camera MDX503 (Lanoptik, China). Detection of the morphological characteristics of the third-instar larvae was performed according to Sukontason *et al*., 2004.

### Fly bioassays

#### Preparation of *S. collinsiae* root extract for the determination of larvicidal activity

A stock solution of the ethanolic extract of *S. collinsiae* roots at a concentration of 1600 mg/ml was prepared by dissolving the extract in a mixture of propylene glycol and water. The stock solution was serially diluted twofold to cover a range of 6.25–1600 mg/ml for the direct contact method, while twofold serial dilutions of the extract mixture in the concentration range of 400–1600 mg/ml were prepared for use in the indirect contact method. The problems of solubility of the extract and high viscosity of the extract solution occurred when an extract concentration greater than 1600 mg/ml was prepared. The extract did not homogenously dissolve in solvent, and the viscosity directly increased. Thus, 1600 mg/ml was used as the maximum concentration in this study.

#### Direct contact toxicity test (topical application method)^22^

Third-instar larvae (20) were transferred to a disposable plastic petri dish (90 × 15 mm). Each concentration of the extract solution (2 µl) was directly dropped on the dorsal side of the third-instar larvae of *M. domestica* using a hand microapplicator (Burkard Manufacturing Co. Ltd., England), while 5 µl of the extract mixture was used for the third-instar larvae of *C. megacephala*.

*M. domestica* (10–12 mm in length) were smaller than *C. megacephala* (15–16 mm in length), and the body weight of *C. megacephala* was approximately 5–10 times higher than that of *M. domestica*. Thus, a suitable volume for application to each species was determined. Five microliters of the extract mixture were excessive for the larvae of *M. domestica*, and this large amount of applied extract solution was spilled from their bodies. Two microliters of the extract solution did not affect the larvae of *C. megacephala*; all of the larvae survived. Thus, at the same range of concentrations (6.25–1600 mg/ml), the volumes of the extract mixture used in the groups of *M. domestica* (2 µl) and *C. megacephala* (5 µl) were different. All bioassays were performed at 28–30 °C and 65–70% humidity. The appearance in terms of shape and color of the larvae, including irregular signs, was recorded. The number of emergent flies and the number of dead pupae were counted and compared with the acetone-treated group as a negative control group, and the imidacloprid (1% w/v) in acetone solution-treated group was used as a positive control group. All experiments were performed in triplicate.

#### Indirect contact toxicity test (contact toxicity test using filter paper)^23^

Each concentration of the extract solution (1 ml) was dropped on Whatman^TM^ filter paper No. 4 (GE healthcare UK Limited, China) and left in a dark place at 25 ± 1 °C for 24 h. The filter paper was then dried in a hot air oven at 50 ± 1 °C for 4–6 h. The concentrations of the extract mixture (two- and four-fold) were prepared by dropping the extract solution onto filter paper, which were then dried and had solution dropped onto it again. The process was repeated two and four times, respectively. The preparation of the filter papers for the negative and positive control groups was performed in the same manner as the extract-soaked filter papers, but acetone and imidacloprid (1% w/v) in acetone were used instead of the extract.

Third-instar larvae (20) were transferred to a Petri dish containing extract-soaked filter paper. All bioassays were performed at 28–30 °C and 65–70% humidity. Physical appearances, such as the color and shape of the larvae, including signs of gross toxicity, were observed and compared with the negative and positive control groups. All experiments were performed in triplicate.

### Parameters and statistical analysis

The development of third-instar larvae to the pupal stage was observed daily until adult flies emerged from the pupa. The pupal mortality and percentage adult emergence were calculated. Mortality data were corrected using Abbott’s formula^24^. Probit analysis using the program March 1987 version^25^ was performed to calculate median lethal concentrations (LC_50_ values) and lethal concentrations that killed 90% of the larvae (LC_90_ values). Statistical analysis was performed in IBM SPSS statistics Version 28.0.1.1 (15) (Chicago, SPSS Inc.). Normal distribution was checked using Kolmogorov–Smirnov test. Difference of *S. collinsiae* extract-treated group and imidacloprid-treated group was analyzed using independent sample t-test or Mann–Whitney U test based on result of normality. All experimental data were analyzed at the 95% confidence interval.

### Research involving plants

All required approvals were obtained for the study, which complied with all relevant regulations.

## Results

### Direct contact toxicity test (topical application method)

The sizes of the third-instar larvae of *M. domestica* and *C. megacephala* were approximately 10–12 and 15–16 mm in length, respectively. In the range of concentrations tested, 0.0063–0.0500 mg/larva, the larvae of *M. domestica* that were killed had a calculated corrected mortality of 44–92%. At the highest concentration of 73.92 mg/larva, the larvae of *C. megacephala* that were killed had a calculated corrected mortality of 37%. Because the percent of corrected mortality in the *C. megacephala* group was less than 50%, the LC_50_ and LC_90_ values could not be calculated. The LC_50_ and LC_90_ values are shown in Table 1.

**Table 1:** Lethal concentration of *S. collinsiae* root extract of two species of flies.

Fly	LC_50_ ± SD (mean ± SD)		LC_90_ ± SD (mean ± SD)	
	Direct contact (mg/larva)	Contact toxicity using filter paper (mg/cm^2^/larva)	Direct contact (mg/larva)	Contact toxicity using filter paper (mg/cm^2^/larva)
*M. domestica*	0.0064 ± 0.0005	0.0165 ± 0.0002	0.0356 ± 0.0005	0.3926 ± 0.0003
*C. megacephala*	> 73.92	1.0500 ± 0.0001	> 73.92	2.7001 ± 0.0003

LC_50_ = lethal concentration that kills 50% of exposed larvae.

LC_90_ = lethal concentration that kills 90% of exposed larvae.

The experiment was evaluated at the 95% confidence interval.

Larvae of *M. domestica* in the 1% w/v imidacloprid-treated group calculated as 0.001 mg/*M. domestica* larva and 0.0025 mg/ *C. megacephala* larva showed a corrected post- treatment mortality of 98 ± 1%, while the *C. megacephala* presented 90 ± 1% corrected mortality. After directly applying 1% w/v of 70% ethanolic *S. collinsiae* root extract calculated as 0.0250 mg/*M. domestica* larva and 0.0625 mg/ *C. megacephala* larva, the groups of *M. domestica* and *C. megacephala* larvae exhibited 92 ± 1 and 5 ± 1% corrected mortality, respectively. At the concentration, percentage of corrected mortality in group of *M. domestica* receiving the extract was significantly different from the 1% w/v imidacloprid-treated group at *p* < 0.001. Small content of imidacloprid significantly presented larvicidal activity greater than *S. collinsiae* extract.

**Figure 1:**
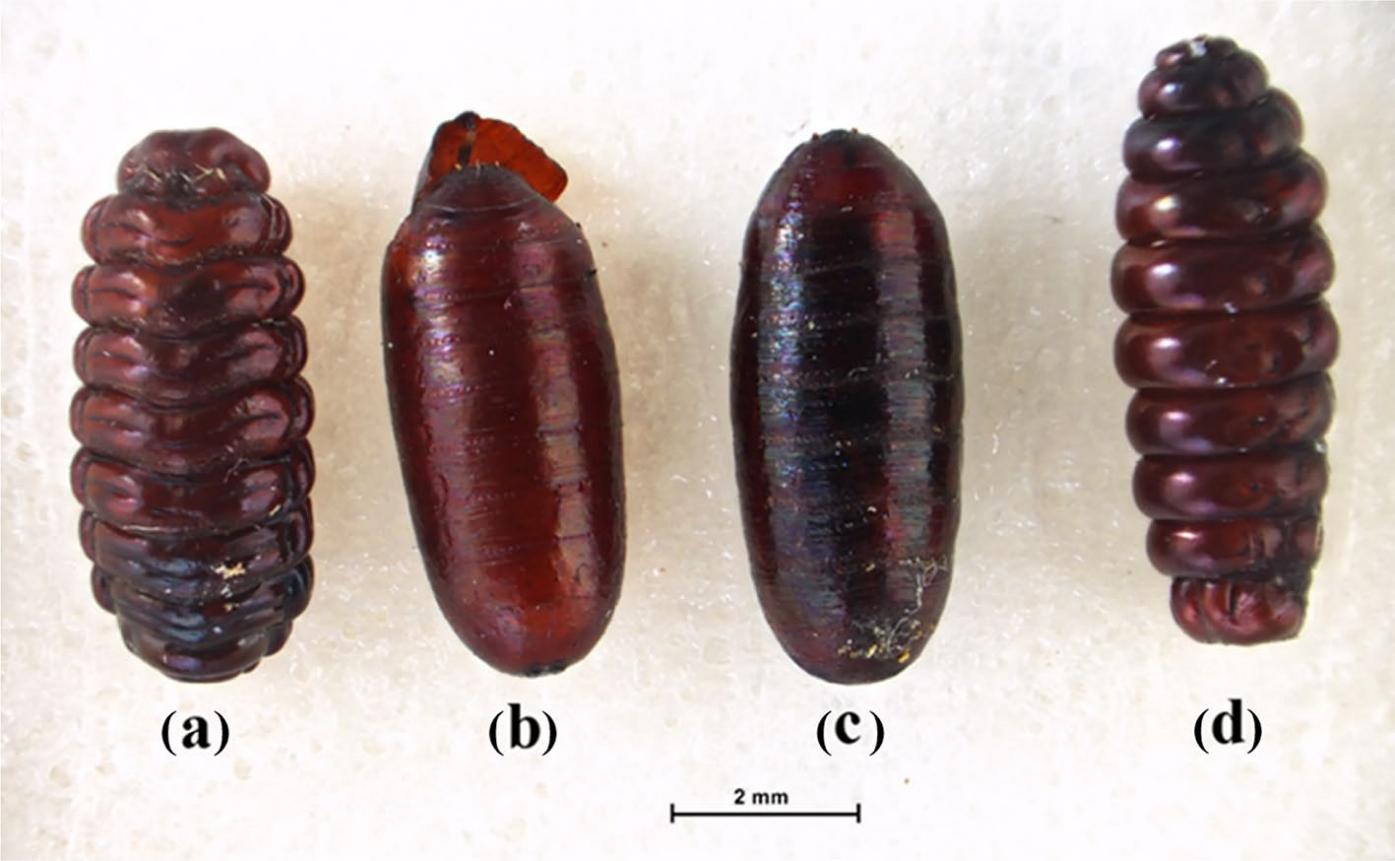
Several pupa forms of *M. domestica* tested with the direct contact method. (a) Segmental pupa in the *S. collinsiae* extract-treated group; (b) normal pupa with capping at the end; (c) normal pupa containing a developing fly; and (d) segmental pupa in the imidacloprid-treated group.

**Figure 2:**
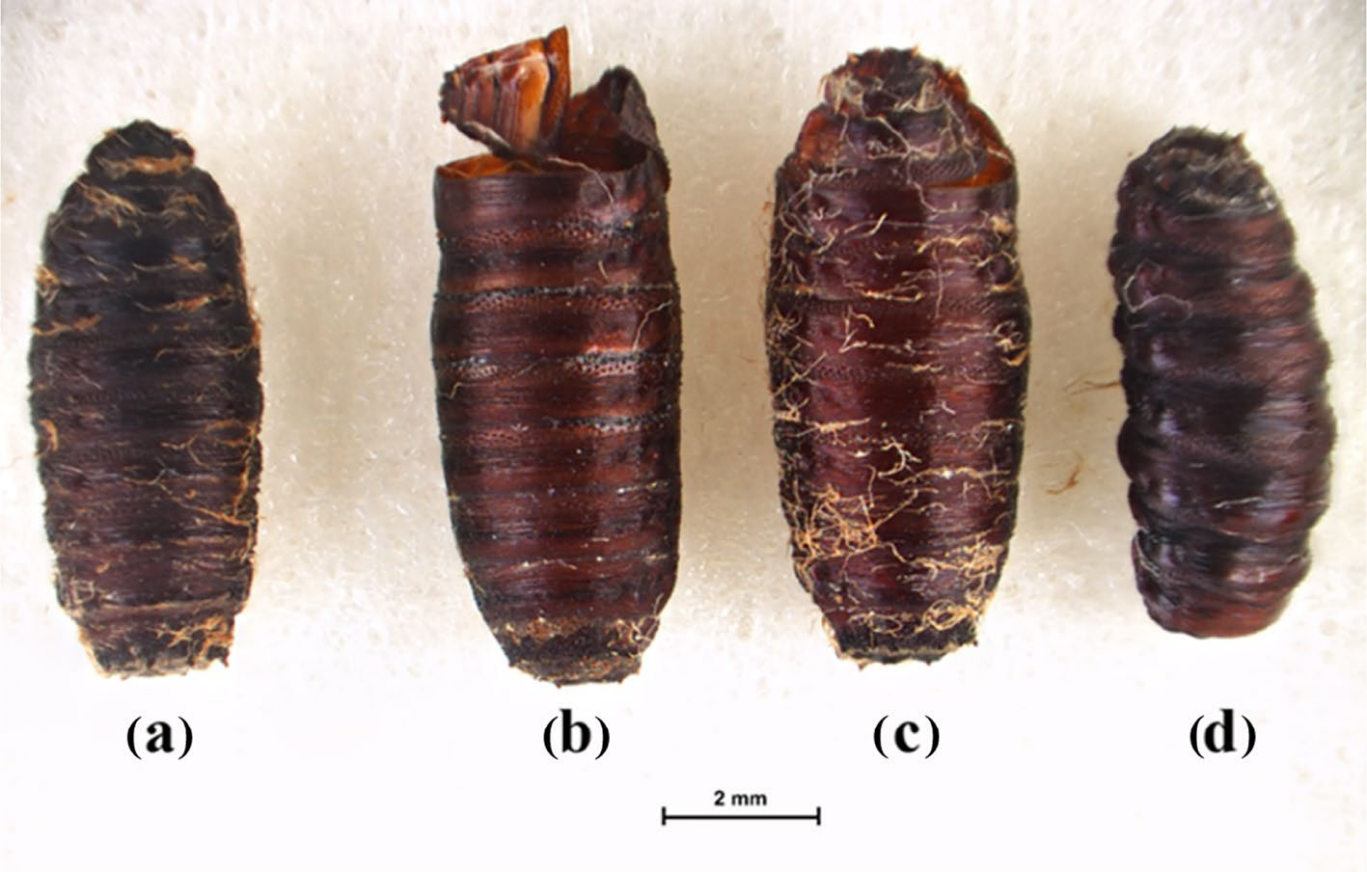
Several pupa forms of *C. megacephala* tested with the direct contact method. (a) Slightly segmental pupa in the *S. collinsiae* extract-treated group; (b) and (c) normal pupa with capping at the end; and (d) segmental pupa in the imidacloprid-treated group.

Segmental puparia with a curved shape were clearly observed in the group of *M. domestica* treated with the *S. collinsiae* root extract, while the pupae in the acetone-treated group were round at both ends and had a barrel-like shape. The same irregular characteristics occurred in the puparium in the *C. megacephala*. The abnormal characteristics of the puparium of *M. domestica* and *C. megacephala* are shown in Figs. 1 and 2.

After coming in direct contact with the *S. collinsiae* root extract, irregular symptoms were initially expressed in the third-instar larvae of both species of flies as a quick movement around the lid of a petri dish followed by motionlessness, contraction of the body with quick mouthpart movements, including the contraction of the mouthpart into the body and glittering of the body due to the production of a cuticle for the puparium. The pupation process of the larvae in the acetone-treated group occurred continuously for 24 h, whereas the pupation process of the *S. collinsiae* extract-treated group occurred quickly (approximately 7 h). In the group of larvae of both flies receiving acetone, adult flies emerged from normal-shaped pupae (0–5% observed mortality), whereas in the groups of larvae receiving the *S. collinsiae* root extract and imidacloprid, adult flies did not emerge from the irregular pupae. Both species of larvae in the imidacloprid-treated group expressed irregular symptoms similar to the group of larvae receiving the *S. collinsiae* root extract.

### Indirect contact toxicity (Contact toxicity using filter paper)

At concentrations in the range of 0.0025–0.6285 mg/cm^2^/larva *S. collinsiae* root extract, the larvae of *M. domestica* that were killed had a calculated corrected mortality of 23–94%. The 23–95% corrected mortality of the larvae of *C. megacephala* was in the concentration range of 0.3967–3.1740 mg/cm^2^/larva. The LC_50_ and LC_90_ values are shown in Table 1.

The larvae of *M. domestica* and *C. megacephala* in the 1% w/v imidacloprid-treated group calculated as 0.0079 mg/cm^2^/larva showed post-treatment mortality of approximately 98 ± 1 and 90 ± 1 percent of the corrected mortality, respectively. The larvae of *M. domestica* which exposed to filter paper soaked the 1% w/v extract solution, calculated as 0.0098 mg/cm^2^/larva, showed post-treatment mortality at 53 ± 2% corrected mortality, while *C. megacephala* larvae were not killed (0 ± 0% corrected mortality). At the concentration, percentage of corrected mortality in group of *M. domestica* receiving the extract was significantly different from the 1% w/v imidacloprid-treated group at *p* < 0.001. Imidacloprid with small content significantly presented larvicidal activity greater than *S. collinsiae* extract.

**Figure 3:**
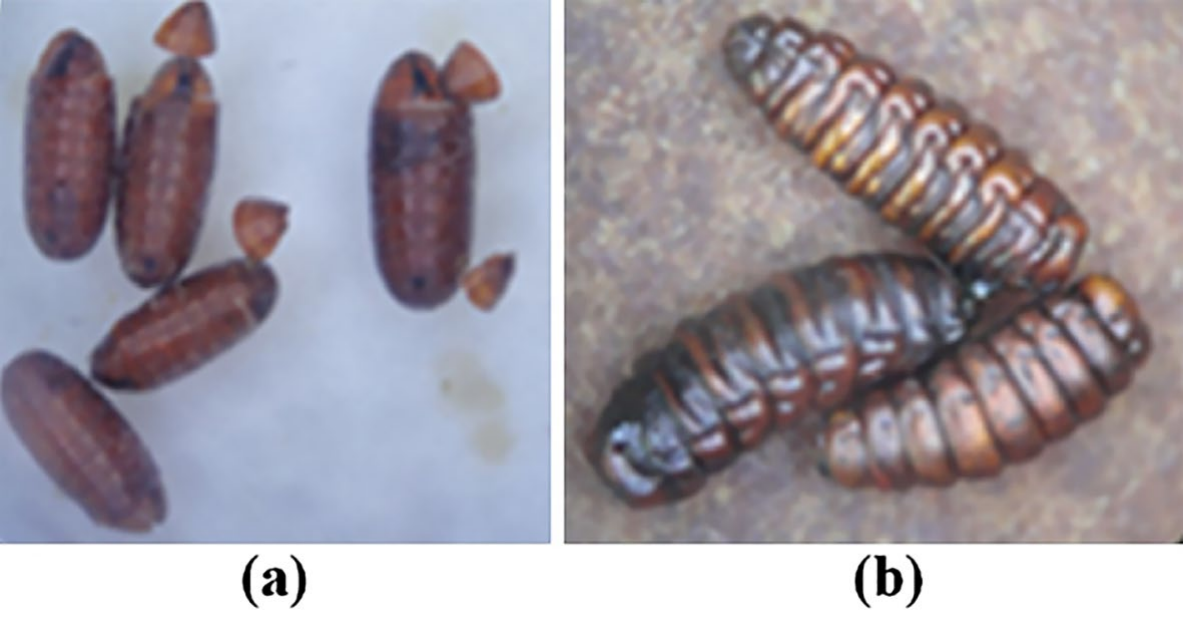
Comparison of the negative control group and the *S. collinsiae* root extract-treated group using the secondary contact method. (**a**) Acetone-treated group and (**b**) *S. collinsiae* root extract-treated group.

The larvae of *M. domestica* that contacted with the extract-soaked filter paper developed into pupae that were brownish black in color with a segmented puparium, curved shape, and pupae that were not round at both ends. The characteristics of the abnormal pupae are shown in Fig. 3b. The larvae in the imidacloprid-treated group displayed the same appearance as the larvae in the extract-treated group. Moreover, the larvae of *M. domestica* in the acetone-treated group developed normally into a barrel-like shape (Fig. 3a), and adult flies were able to completely emerge from the pupae.

### Detection of the phytochemicals in the 70% ethanol *S. collinsiae* crude extract using TLC

**Figure 4:**
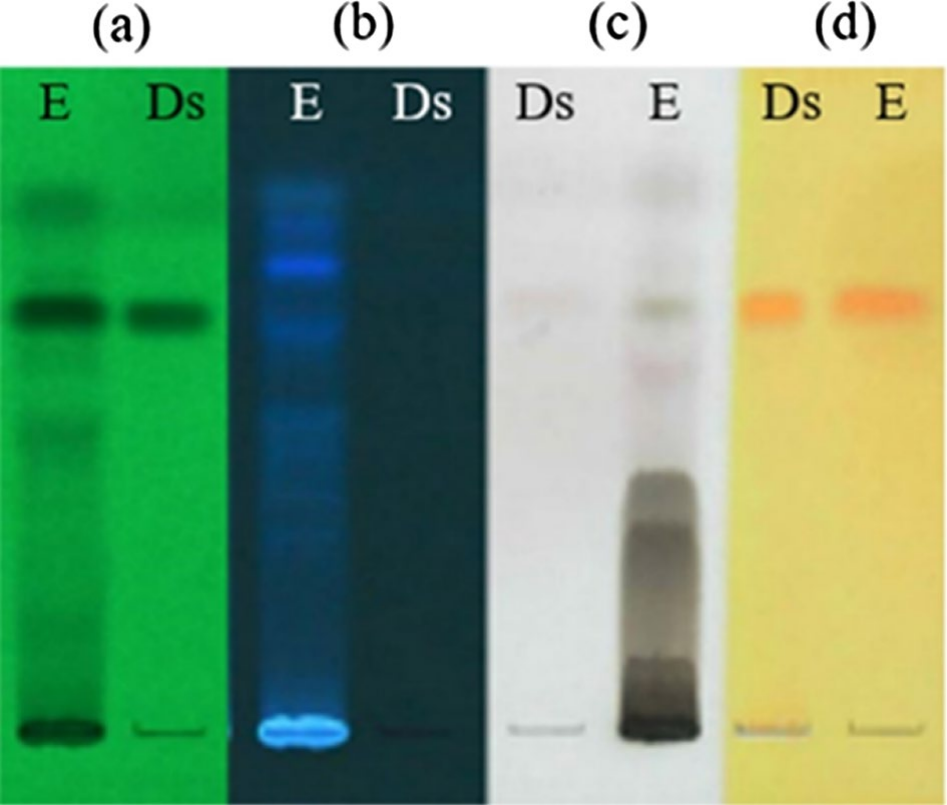
The TLC fingerprint of the *S. collinsiae* root extract was observed under UV light at (**a**) 254 nm and (**b**) 366 nm, (**c**) after spraying with 10% H_2_SO_4_ and then heating, and (**d**) after spraying with Dragendorff’s spray reagent. E represents the 70% ethanol *S. collinsiae* root extract, and Ds represents the didehydrostemofoline reference substance (hRf = 62.5).

Compared with the band of the didehydrostemofoline reference substance (hR_f_ = 62.5) (Fig. 4, Track Ds), a quenching band of didehydrostemofoline, observed under UV light at 254 nm, was clearly found in the 70% ethanol *S. collinsiae* crude extract (Fig. 4a, Track E). After spraying 10% H_2_SO_4_ and then heating, a black band from didehydrostemofoline appeared (Fig. 4c, Track E), while an orange band from didehydrostemofoline was observed after spraying with Dragendorff’s spray reagent (Fig. 4d, Track E). All bands had the same hR_f_ of 62.5 (Fig. 4), which indicated the presence of the alkaloid didehydrostemofoline in the 70% ethanol *S. collinsiae* crude extract. When observed at 366 nm (Fig. 4b), bands of unknown blue fluorescent substances were obviously seen. The alkaloid didehydrostemofoline and unknown blue fluorescent substances were dominant in the TLC fingerprint of the 70% ethanol *S. collinsiae* root extract (Fig. 4).

## Discussion

The third-instar larvae of *M. domestica* and *C. megacephala* were used in this experiment because third-instar larvae are the final stage of larval development. The third-instar larvae are different from the first- and second-instar larvae in terms of their size being the largest, and complete spiracle and other organs, especially the posterior spiracle, which is used for identification^26,27^. The lowest LC_50_ and LC_90_ values were found in the groups of *M. domestica* using direct and indirect contact methods while the LC_50_ and LC_90_ values of *C. megacephala* could not be determined by the direct or indirect contact toxicity method. The *S. collinsiae* root extract was able to kill both *M. domestica* and *C. megacephala* in the larval stage after dermal administration, while *M. domestica* was clearly killed by both direct and indirect contact. The sensitivity of *M. domestica* to the *S. collinsiae* extract was greater than that of *C. megacephala*. The elimination of *M. domestica* using *S. collinsiae* root extract was easier than *C. megacephala* because the size and weight of *M. domestica* larvae were approximately one-fold less than those of *C. megacephala*. The concentration of *S. collinsiae* root extract used to kill *C. megacephala* was higher than that of *M. domestica* by approximately 100-fold and tenfold for direct and indirect contact toxicity, respectively. The larvae of *C. megacephala* were successfully killed via indirect contact toxicity due to the 200-fold higher concentration of the *S. collinsiae* root extract used in the indirect contact method than in the direct contact method. The larvae frequently contacted the filter paper with the absorbed extract. Thus, the low concentration of *S. collinsiae* root extract was suitable to eliminate *M. domestica*. To eliminate *C. megacephala* via direct contact administration, a high concentration of the extract should be used.

A brownish black color, segmental puparia, and curved shape were observed for both species of flies after direct and indirect topical administration of the extract, but these irregular appearances appeared more frequently in the pupa of *M. domestica* than in the pupa of *C. megacephala*. Normally, the puparia of *M. domestica* and *C. megacephala* are coarctate and cylindrical in shape with both ends being round^28^. The expression of these irregular characteristics was similar to the characteristics of the larvae in the imidacloprid-treated group. The larvae of both flies in the acetone-treated group did not show these abnormal appearances or characteristics. The adult flies were able to emerge from pupae and develop into complete adult flies. The larvae that were treated with the *S. collinsiae* root extract using the indirect contact method showed the same symptoms and physical appearances as the larvae in the group that received a direct drop of the *S. collinsiae* extract on the body. The *S. collinsiae* root extract also affected the larval development of *Parasarcophaga ruficornis*^8^. Moreover, *S. collinsiae* root extract exhibited insecticidal activity against various insects. A study of the acaricidal activity of the nine species of *Stemona* against *Rhipicephalus microplus* found that *S. collinsiae* root extract had the highest activity and significantly reduced the attachment of ticks to calf skin^29^. Neonate larvae of *Spodoptera littoralis* were eliminated by the root extract of *S. collinsiae* and didehydrostemofoline, which displayed stronger insecticidal activity than the root extract of *S. tuberosa*^6^. Third-instar larvae of the pyrethroid-resistant diamondback moth (*Plutella xylostella*) were killed by 16,17-didehydro-16(E)-stemofoline, which was isolated from the methanolic root extract of *S. collinsiae*^4^. Additionally, the roots of *S. collinsiae* contained a higher content of insecticidal *Stemona* alkaloids, especially didehydrostemofoline, than other species of *Stemona*^30^. Thus, it was possible that these alkaloids were active compounds that could kill *M. domestica* and *C. megacephala* larvae. In accordance with the TLC fingerprint of the 70% ethanol *S. collinsiae* extract, a large amount of didehydrostemofoline was predominant in the extract. Unknown blue fluorescent substances may be rotenoid flavonoids, such as stemonal^31,32^, stemonacetal, and stemonone^32^ or stilbenoids, such as stemofurans, dihydrostilbenes, stilbostemins, pinosylvin, dihydrophenanthrene racemosol^33^. Didehydrostemofoline possesses inhibitory activity of acetylcholinesterase^34,35^. A molecular docking study revealed that stemonal could strongly interact with the catalytic site of acetylcholinesterase and showed anti acetylcholinesterase activity^36^. The cholinergic system and acetylcholine influence the secretion of insect hormones, such as juvenile hormone, prothoracicotropic hormone, and ecdysone hormone, larval development, pupation, and eclosion^37^, including the growth and function of the hypopharyngeal gland^38^. Acetylcholine and acetylcholine agonists can induce the secretion of prothoracicotropic hormones from neurosecretory cells^39,40^. Prothoracicotropic hormone stimulates the secretion of ecdysteroid from the prothoracic gland, and pupation, the alteration of body color and the production of new cuticles can be induced. Moreover, juvenile hormone regulates larval-larval development and is released slightly during pupation^41^. In our research, pupation of *M. domestica* and *C. megacephala* receiving a mixture of the 70% ethanol *S. collinsiae* root extract was accelerated, compared with the negative control group. The premature pupae with an irregular shape was possibly caused by the alkaloid didehydrostemofoline and rotenoid stemonal containing in the extract, because these compounds possess inhibitory activity of acetylcholinesterase. The inhibition of acetylcholinesterase induced the accumulation of acetylcholine in the pharate larvae. The accumulated acetylcholine may stimulate the release of prothoracicotropic hormone and ecdysteroid and contraction of the body muscles with the production of a new cuticle. Thus, segmental pupae were produced. Signs of toxicity presenting in the *S. collinsiae* extract treated group were similar with the group receiving imidacloprid solution. Imidacloprid, neonicotinoids, is classified as a nicotinic acetylcholine receptor competitive modulator^42^. When cholinergic system was stimulated, the secretion of juvenile hormone and pupation and organogenesis were interrupted ^43–45^. However, the 70% ethanol *S. collinsiae* root extract contained a mixture of insecticidal alkaloids and rotenoid flavonoids including stilbenoids. Insecticidal rotenoids inhibit mitochondrial NADH dehydrogenase (Complex I), and the electron transport chain is suppressed^42^. Recently, insecticidal activity of stilbenoids was reported. For example, stilbenoids extracted from *Vitis vinifera* canes, possessed insecticidal activity against *Spodoptera littoralis* larvae^46^. (Z)-2-arylstilbenes showed high potency of insecticidal activity against *Spodoptera exigua*, *Trichoplusia* ni, *Helicoverpa zea*, *Plutella xylostella* and *Pseudoplusia includes*^47^. Lignans and stilbenoids acted on insect growth regulation^48^. Oligostilbenes competed with ecdysteroid to bind to dipteran and lepidopteran ecdysteroid receptors^49^. Also, pinosylvin, stilbenoid disturbed ecdysis^48^, was found in methanolic *S. collinsiae* extract^33^. Impairment of larval-pupal development was possible caused from stilbenoids in *Stemona* plant. But it was unclear and should be tested in the further experiment. Insecticidal activity of the *S. collinsiae* root extract was occurred from synergistic of several natural insecticides, described in Phayakkaphon *et al*^50^. The *S. collinsiae* root extract dominantly displayed neurotoxic via inhibition of acetylcholinesterase and stimulation of acetylcholine neurotransmitter. Didehydrostemofolien and other *Stemona* alkaloids possible induced function of cholinergic system. Firstly, fast movement of the larvae was occurred and followed by pupation with contraction of body muscle. Action of the cholinergic system related to secretion of hormone regulating metamorphosis of *M. domestica* and *C. megacephala*. Irregular pupation and abnormal morphology of puparia were expressed after received *S. collinsiae* root extract. But, mechanism of action of didehydrostemofoline and other *Stemona* alkaloids was unclear. The insecticidal activity of isolated *Stemona* alkaloids, rotenoids and stilbenoids should be tested in the further experiment for more clarify.

At concentration 1% w/v of 70% ethanolic *S. collinsiae* root extract calculated as 0.0250 mg/*M. domestica* larva and 0.0625 mg/ *C. megacephala* larva, *C. megacephala* larva received content of the extract higher than *M. domestica* larva about 2.5 times. The 70% ethanolic *S. collinsiae* root extract presented 92 ± 1 and 5 ± 1% corrected mortality against third-instar larvae of *M. domestica* and *C. megacephala* via direct contact administration, respectively. Susceptibility results of the *S. collinsiae* root extract against *M. domestica* larvae using direct contact route was interpreted in level of possible resistance (90–97% mortality)^51^ while elimination of *C. megacephala* was classified in confirmed resistance level (< 90% mortality)^51^. *M. domestica* larvae could be greatly eliminated by the *S. collinsiae* root extract via direct contact method while almost of *C. megacephala* larvae was survived. Meanwhile, *M. domestica* larvae possible resisted to the *S. collinsiae* root extract.

For indirect contact route, the 1% w/v of *S. collinsiae* root extract calculated as 0.0098 mg/cm^2^/larva exhibited elimination of *M. domestica* and *C. megacephala* larvae at a corrected mortality of 53 ± 2% and 0 ± 0%, respectively. The presence of 0 ± 0% corrected mortality happened in group of *C. megacephala* larvae because the used concentration of *S. collinsiae* root extract as 0.0098 mg/cm^2^/larva was lower than the tested concentration range of 0.3967–3.1740 mg/cm^2^/*C. megacephala* larva using for calculation of LC values about 40–300 times. At this concentration (0.0098 mg/cm^2^/larva), susceptibility of *S. collinsiae* root extract against *M. domestica* was considered in level of confirmed resistance^51^. The lowest percent of corrected mortality was obviously presented in *C. megacephala* larvae. The extract did not affect on *C. megacephala* larvae. The indirect contact administration and the concentration of the extract (0.0098 mg/cm^2^/larva) was clearly not suitable for elimination *C. megacephala* larvae.

Comparing with 1% w/v of imidacloprid solution, it possessed a corrected mortality of 98 ± 1% for eliminating *M. domestica* larvae and 90 ± 1% corrected mortality in *C. megacephala* larvae. *M. domestica* larvae were considered susceptible to 1% imidaclopride solution while *C. megacephala* possible resisted to the solution. Concentration of imidaclopride solution and the *S. collinsiae* root extract directly related to larvicidal activity. The concentration of imidaclopride solution may be increased more than 1% for effective elimination of *M. domestica* and *C. megacephala* larvae.

Thus, elimination of *M. domestica* with low content of 70% ethanolic *S. collinsiae* root extract using contact administration was interesting. Concentration of the *S. collinsiae* root extract for *M. domestica* control using direct and indirect contact administration should be more than 0.04 mg/larva and 0.4 mg/cm^2^/larva, respectively. *M. domestica* larvae possible resisted to the *S. collinsiae* root extract. In further experiment, it should be tested with enlarged number of populations and many batches of the *S. collinsiae* root extracts, also field study. For *C. megacephala* control, concentration of the extract should be increased more than 74 mg/larva and 3 mg/cm^2^larva. However, cost-effectiveness should be considered. The larvicidal activity against *C. megacephala* did not exhibit when small content of the extract (0.0625 mg/ *C. megacephala* larva and 0.0098 mg/cm^2^/larva) was used.

Effect of 70% ethanol *S. collinsiae* root extract on larval-pupal development of *M. domestica* and *C. megacephala* via contact routes was reported in this research. 70% ethanolic *S. collinsiae* extract could be used as larvicidal ingredient in liquid, powder or aerosol formulations for fly control in livestock farm and waste disposal.

## Conclusions

In this study, *M. domestica* and *C. megacephala* in the larval stage were killed by *S. collinsiae* root extract after topical administration, similar to *P. ruficornis*. The abnormal symptoms of the larvae receiving the extract were likely a result of the administration of the *Stemona* alkaloids and imidacloprid. The extract contained insecticidal didehydrostemofoline and unknown blue fluorescent substances. The mechanism of action of the extract is related to anti-acetylcholinesterase activity and acetylcholine modulation by the substances. The accumulated acetylcholine induced the release of prothoracicotropic hormone and ecdysteroid. It affected pupation. The muscles of the larvae contracted with the production of a new cuticle. Irregular segmental pupae were observed. Organogenesis during metamorphosis and the secretion of juvenile hormone were disturbed. Malformations of the adult flies in the pupae occurred until they could not emerge. 70% ethanol *S. collinsiae* root extract was growth regulator via mechanism of acetylcholine system. Concentration of *S. collinsiae* root extract directly associated with larvicidal activity. Low concentrations of *S. collinsiae* root extract were able to terminate the life cycle of medically important flies, especially *M. domestica*, by promoting larvicidal activity. *M. domestica* is found in high numbers in Thailand. Thus, it is interesting to develop products from *S. collinsiae* root extract as an alternative natural insecticide and fly eliminator.

## Data Availability

All data are shown in the manuscript.
